# The Overtreatment and Cost Effectiveness of Primary *versus* Secondary Maintenance Therapy with Poly-Adenosine Ribose Phosphate Inhibitors (PARPi) for Epithelial Ovarian Cancer (EOC)

**Published:** 2021-10-07

**Authors:** Peter G Rose, Meng Yao, Laura M Chambers, Lin Mei, Phuc Le

**Affiliations:** 1 Division of Gynecologic Oncology, Women’s Health Institute, Cleveland Clinic, Cleveland Ohio, USA; 2 Department of Quantitative Health Sciences, Cleveland Clinic, Cleveland, Ohio, USA; 3 Department of Internal Medicine, Cleveland Clinic, Cleveland, Ohio, USA

**Keywords:** Ovarian cancer maintenance, Olaparib, Niraparib Cost/Efficacy, BRCA mutation status

## Abstract

**Background:**

No data exist to suggest PARP inhibitor (PARPi) therapy as first-line maintenance is superior to PARPi therapy as second-line maintenance.

**Objective:**

To determine the efficacy and cost of primary versus secondary olaparib or niraparib maintenance in Epithelial Ovarian Cancer (EOC).

**Methods:**

A retrospective cohort study was performed in women with EOC to determine the survival following primary or secondary PARPi maintenance. We modeled the costs of olaparib and niraparib based on previously published costs and duration of therapy based on the Solo 1/Solo 2 and Prima and Nova trials, respectively.

**Results:**

Among 40 patients treated with PARPi as primary or secondary maintenance there was no difference in overall survival (p=0.97). Among 166 women with stage III/IV germ-line BRCA mutated EOC, 28.8% were disease free for >3 years (18.6% never recurred and 10.2% recurred >3 years after chemotherapy). Since 29% of the BRCA mutated patients did not recur within 3 years, primary olaparib maintenance therapy was significantly more expensive than secondary olaparib maintenance therapy by 260%. Primary niraparib maintenance therapy was slightly more expensive than secondary niraparib maintenance therapy by 4%, 51%, and 15% for BRCA mutated, HR deficient, and HR proficient patients, respectively. By eliminating the overtreatment of patients with primary PARPi therapy, the cost savings for 100 women with EOC with BRCA mutations would be $37,335,360 for olaparib and $8,197,592 for niraparib.

**Conclusion:**

Up to 29% of BRCA mutated patients may be overtreated with primary PARPi maintenance with significantly increased treatment costs.

## INTRODUCTION

Inhibitors of Poly-Adenosine Ribose Phosphatase (PARPis) have recently gained broad approval in the treatment of epithelial ovarian, peritoneal, and Epithelial Ovarian Cancer (EOC). While PARPi was initially approved only for patients with germline and subsequently somatic BRCA mutation, current approvals allow for PARPi as primary maintenance therapy following first-line chemotherapy and as secondary maintenance following platinum-sensitive recurrence, irrespective of BRCA mutational status. Cost and overall survival with PARPis are the primary concerns for patients with EOC [1].

To date, limited data is available about the impact of PARPis on overall survival in EOC. Additionally, there is currently no data that PARPi use as primary maintenance therapy is superior to the use of these agents at the time of recurrence. Notably, the small nonsignificant survival benefit with olaparib seen in Study 19, which evaluated secondary olaparib maintenance in platinum-sensitive recurrent EOC, only became statistically significant when patients who subsequently received PARPi were excluded from the data analysis [2]. This suggests that secondary therapy may be as effective as primary therapy. Recently, SOLO2 reported an improvement in overall survival with the use of secondary olaparib maintenance in platinum sensitive BRCA mutated patients with recurrent disease compared to placebo, HR 0.74 (0.54-1.00) [3]. Unfortunately, only 38% of patients in the control arm ever received subsequent therapy with a PARPi, which may have negatively affected their potential for survival. Notwithstanding this, the improvement in survival noted in the above trial demonstrates that these agents are beneficial for the treatment of EOC. However, the recently updated NOVA trial reported no statistical improvement in overall survival with the use of niraparib following platinum-sensitive recurrence in either BRCA mutated (45.9 months *vs* 43.2 months) or non-mutated cohorts (39.1 months *vs* 38.5 months) versus placebo controls [4]. This finding occurred during a period of time in which FDA approval of both olaparib and niraparib allowed potential crossover, however, the trial did not monitor subsequent therapy.

However, one of the important questions facing oncologists is whether patients with EOC should be treated with a PARPi following primary chemotherapy or following secondary chemotherapy at the time of recurrence or later. There are a number of factors that one may consider for delaying treatment with PARPi until the time of recurrence including avoiding overtreatment, quality of life, cost, toxicity, and overall survival. Since no randomized study has compared primary versus secondary PARPi maintenance therapy in the treatment of EOC, no level I evidence exists to evaluate these factors. In this study, we have elected to look at the differences between the use of PARPi for either primary maintenance or secondary maintenance in platinum-sensitive recurrent ovarian cancer focusing on efficacy, overtreatment, and cost.

## METHODS

### Data collection

This was an IRB approved (IRB-7419) retrospective cohort study in women diagnosed with EOC in the Cleveland Clinic Health System. Informed consent was waived by the IRB. Electronic medical records were reviewed to identify treatment modalities, BRCA mutational status, Progression Free (PFS), and overall survival. To determine the frequency and timing of recurrence, comparison groups of women with (1) germ-line or somatic BRCA mutated EOC and (2) non-BRCA mutated EOC from 2009-2015 were analyzed.

### Efficacy

The overall survival of EOC patients treated with a PARPi after first-line or second-line platinum-based chemotherapy was compared.

### Cost

A decision analysis model was created to compare the cost of primary or secondary maintenance PARPi therapy. Both olaparib and niraparib are currently the only FDA approved drugs that can be used as maintenance therapy in both the primary and platinum-sensitive recurrent patient populations. In this analysis the cost of primary and secondary maintenance was evaluated for olaparib in patients with germline BRCA mutations and for niraparib in the germline BRCA mutated, tumor Homologous Recombinant Deficient (HRD) positive, and HRD negative populations. In the Solo I trial, which assessed olaparib maintenance after first-line chemotherapy for BRCA mutated patients, the PFS was 49.9 *versus* 13.8 months for the treatment versus placebo groups, respectively [5]. Table 1 in Solo 2, which assessed olaparib maintenance after second-line platinum sensitive chemotherapy for BRCA mutated patients the PFS was 19.1 versus 5.5 months for the treatment versus placebo groups, respectively [6]. In the PRIMA trial, which assessed niraparib maintenance after first-line chemotherapy, the PFS survival for BRCA mutated patients was 22.1 versus 10.9 months, for HR deficient patients 19.6 versus 8.2 months and for HR proficient patients 8.1 versus 5.4 months [7]. In the NOVA trial, which assessed niraparib maintenance after second-line platinum sensitive chemotherapy, the PFS for BRCA mutated patients was 21 *versus* 5.8 months, for HR deficient patients 12.9 versus 3.8 months and for HR proficient patients 9.3 versus 3.9 months for treated and control groups, respectively [8]. Therefore, these durations of treatment were chosen to determine the costs of maintenance therapy. Since SOLO 1 reported PFS at 36 months following registration, the timing of recurrence in our patient population was carefully analyzed [5].

Costs associated with the use of olaparib or niraparib were calculated using the previously published decision cost analysis by Zhong, et al. [9]. Their study found costs associated with the use of olaparib or niraparib versus observation included: the cost of the drug, the cost of physician visits, the cost of additional lab work and imaging studies. CT scans of the chest abdomen and pelvis were obtained every 3 months for patients on olaparib or niraparib and yearly for patients who were in observation. The cost of genetic screening for patients with ovarian cancer was not included as this is recommended for all patients with ovarian cancer by the American Society of Clinical Oncology. Their study found the annual cost for olaparib or niraparib was $123,200 or $138,000, respectively, while the annual cost for observation was $1,200. All costs were reported in 2017 US dollars. The total cost for olaparib or niraparib was calculated based on the median time patients were on therapy in Solo 1/Solo 2 or Prima/Nova trial, respectively.

To ensure costs were calculated across subgroups of treated and observed patients equally a strict treatment paradigm was utilized. Patients who completed first-line chemotherapy and were treated with olaparib or niraparib maintenance in the front line setting and progressed would be subsequently treated with a platinum combination (carboplatin and paclitaxel, carboplatin and gemcitabine, or carboplatin and pegylated liposomal doxorubicin). Patients who completed first-line chemotherapy and were observed and progressed would be subsequently treated with a platinum combination (carboplatin and paclitaxel, carboplatin and gemcitabine, or carboplatin and pegylated liposomal doxorubicin) before olaparib or niraparib maintenance. The approach of using platinum primarily in platinum sensitive patients is supported by the randomized trial MITO-8 [10]. The costs of second line chemotherapy were not calculated as this is considered standard of care.

## RESULTS

Four hundred and eighty-two patients with stage III and IV EOC were included in this analysis. One hundred and sixty-six were germ line or somatic BRCA mutated, 193 were BRCA negative and 123 were not tested for BRCA mutations. Somatic mutation analysis was not routinely performed.

### Effect of primary or secondary PARPi on survival

**Figure 1:**
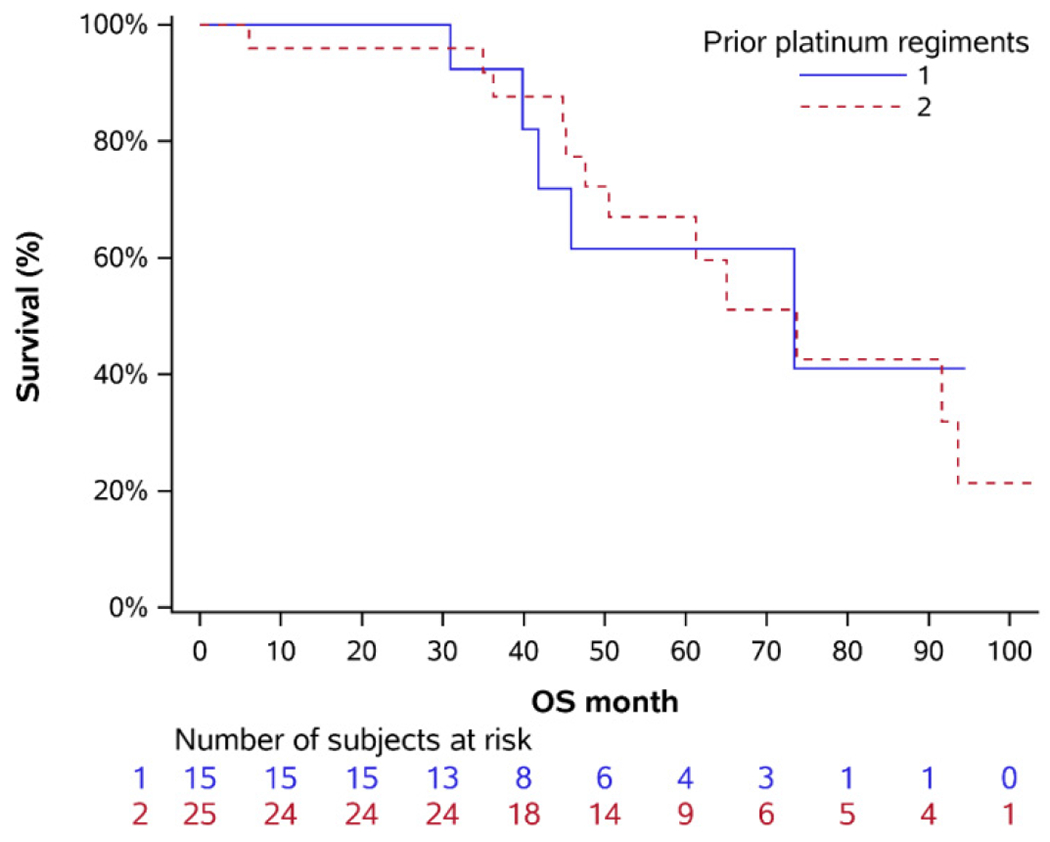
Overall survival following primary or secondary PARPi maintenance.

We analyzed the survival of 40 patients treated at our institution with either primary or secondary maintenance PARPi therapy. The survival of these patients was not statistically different (p=0.97) and this was further corroborated when controlling for BRCA mutational status p=0.31 and p=0.45 for BRCA positive and BRCA negative, respectively (Table 2, Figure 1).

### Avoiding overtreatment

Among 166 women with FIGO 2014 stage III/IV germ-line BRCA mutated who were followed for greater than 3 years following chemotherapy without PARPi therapy, 31 patients (18.6%) never recurred (median follow-up 8 years, range 3.25-34 years) and an additional 17 patients (10.2%) recurred more than 3 years after their chemotherapy (median 5.1 years, range 3.1-9.5 years). Collectively, 28.8% of stage III and IV patients without PARPi therapy were disease-free for more than 3 years after completing chemotherapy.

Among 193 patients without a BRCA mutation, 39 (20.2%) did not recur and 12 (6.2%) recurred more than 3 years after their chemotherapy (median 4.8 years range 4.3-6.1 years). Among 123 patients never tested for a BRCA mutation, 22 (17.8%) did not recur and 3 (2.4%) recurred more than 3 years after their chemotherapy (median 5.4 years range 4.9-5.5 years). Patients who underwent primary cytoreductive surgery and adjuvant chemotherapy had a lower recurrence rate 72.7% than patients treated with neoadjuvant chemotherapy and interval debulking surgery 82.8%.

### Cost

When we compared the cost of the strategies of primary versus secondary treatment with olaparib for BRCA mutated EOC patients we found a significant difference in cost. Patients who received primary maintenance therapy with olaparib incurred a cost of $512,857, while those who received secondary maintenance therapy with olaparib incurred a cost of $197,473. This large difference was likely due to the significant differences in progression free survival comparing primary and secondary maintenance with olaparib. In contrast when we compared the cost of the strategies of primary versus secondary treatment with niraparib, the total cost is almost identical for EOC patients who have BRCA mutations with total costs of $254,700 and $242,590, respectively (Table 3). In women with recurrent EOC whose tumors are HR deficient, primary treatment resulted in significantly increased cost at $225,780 compared to $149,170 for secondary treatment. For ovarian cancer patients whose tumors are HR proficient primary treatment was only slightly more expensive than secondary treatment at $107,490 and $93,540, respectively.

If we elected not to initiate primary maintenance for BRCA mutated ovarian cancer patients, 28.8% of patients may not recur or recur late resulting in significantly decreased costs. The cost for treating 100 BRCA mutated ovarian cancer patients with primary maintenance olaparib would be $51,285,700 versus $14,060,078 for secondary maintenance representing a cost savings of $37,225,622. The cost for treating 100 BRCA mutated ovarian cancer patients with niraparib would be $25,470,000 versus $17,272,408 representing a cost savings of $8,197,592.

## DISCUSSION

In this study, we evaluated the efficacy and modeled the cost of primary versus secondary maintenance therapy with a PARPi in women with EOC based on previous publications. We found that our patients treated with primary or secondary PARP inhibitor maintenance had the same survival. We did not study cost-effectiveness because Study 19 and our own data suggest incremental cost of earlier maintenance therapy does not result in improved survival [11].

In our model of evaluating primary treatment with niraparib followed by observation versus observation followed by treatment with niraparib there is no significant difference in the time to the second recurrence. However, olaparib as primary maintenance therapy was associated with a significant improvement in PFS which was not achieved with olaparib as secondary maintenance therapy. This may be due to significant differences in the makeup of the Solo 1 and Prima trials. Specifically, the Solo 1 trial had a lower percentage of patients (15%) with stage IV disease than the Prima trial (34.7%). Additionally, primary surgery was used significantly more in the Solo 1 trial (61.9%) versus the Prima trial (36.8%).

While the vast majority of patients with advanced-stage ovarian cancer do recur, among 7651 patients with stage III or IV ovarian cancer enrolled in GOG trials, 17.6% of patients did not recur [12]. However, these trials did not collect information regarding germline or somatic BRCA mutation. Progression-free survival is significantly longer for patients who have BRCA mutations, particularly BRCA2 [13]. In the current study, we found that among patients with BRCA mutations and stage III and IV ovarian cancer 18.6% never recurred, and an additional 10.2% recurred more than 3 years after completing chemotherapy. Collectively, 28.8% did not recur or recurred later than the endpoint of Solo 1. This is nearly identical to reports by Bookman et al. [14] and Jorge et al. [15] who both reported 30% of BRCA patients not recurring. Among stage III and IV ovarian cancer patients who tested negative for a BRCA mutation or who were not tested 19.3% never recurred and an additional 4.7% recurred more than 3 years after completing chemotherapy. Given the potential for both overtreatment and premature treatment our data suggest that the secondary strategy may be preferred.

Strengths of our study include the fact that the duration of therapy used to calculate costs of olaparib and niraparib maintenance therapy is based on four large randomized ovarian cancer maintenance trials. Additionally the costs of the observation arm relies on the previous publication by Zhong et al. which included the cost of the drug, the cost of physician visits, the cost of the additional lab work and imaging studies [9]. We are accepting the assumption that the benefit of chemotherapy is not affected by prior PARP inhibitor therapy. However, recently a retrospective study evaluating the use of olaparib as maintenance therapy in BRCA mutated ovarian cancer patients demonstrated very poor subsequent response to chemotherapy [16]. However, little was provided regarding the type of chemotherapy utilized in this patient population and the authors concluded their data was provocative and need to be confirmed by further studies investigating chemotherapy following PARP inhibitor exposure.

## LIMITATIONS

Weaknesses of the efficacy analysis include the inherent biases in a retrospective study, the non-randomized assignment to treatment and small number of patients analyzed.

The findings of this study underscore the need for a randomized trial evaluating the primary *vs* secondary use of the PARP inhibitors as maintenance therapy. Such a trial should stratify for BRCA mutational status, homologous recombinant deficiency and homologous recombinant proficiency.

Overall survival would be the primary endpoint but toxicity and costs would need to be included.

Our study did find that the costs of primary PARP inhibitor maintenance treatment for BRCA mutated patients is substantially more expensive than secondary PARP inhibitor maintenance treatment since 28.8% of Stage III/IV BRCA mutated patients can avoid overtreatment. Two recent articles suggest that first-line maintenance therapy with olaparib or niraparib are cost-effective but both studies estimate the cost of observation considerably higher at 78% [17] and 28% [18] of the PARPi therapy costs, respectively. However, the previous publication by Zhong et al. [9]. estimated the cost of observation to represent less than 1% of therapy with olaparib or niraparib. In light of the fact that a large percentage of stage III/IV ovarian cancer patients with BRCA mutations never recurred or recurred late, delaying maintenance therapy until the second line setting, results in substantial cost savings.

## Figures and Tables

**Table 1:** Progression free survival in months.

	BRCA Mutated Olaparib	BRCA Mutated Niraparib	HRD^+^ Niraparib	HRD^−^ Niraparib
**Primary treatment OBS** *vs* (**TX**)	13.8 (49.9)	10.9 (22.1)	8.2 (19.6)	5.4 (8.1)
**Secondary treatment TX** *vs* (**OBS**)	19.1 (5.5)	21.0 (5.5)	12.9 (3.8)	9.3 (3.9)
**Total**	32.9 (55.4)	31.9 (27.6)	21.1 (23.4)	14.7 (12)

**Table 2:** Univariate overall survival summary.

Variable	N	Events	Median Months (95% CI)	5-Year Overall % (95% CI)	log-rank p-value
**All patients**	40	18 (45%)	73.4 (47.6-93.6)	65.3 (48.4,82.2)	
	
**prior platinum comparison, all BRCA together**					0.97
	
1	15	5 (33%)	73.4 (39.8)	61.5 (31.5,91.6)	
	
2	25	13 (52%)	73.7 (47.6-93.6)	67.0 (46.7,87.3)	

**prior platinum comparison, only BRCA positive**					0.31
	
1	7	1 (14%)	NA	83.3 (53.5,100.0)	
	
2	13	7 (54%)	91.6 (45.2-124.3)	73.8 (48.2,99.5)	

**prior platinum comparison, only BRCA negative**					0.45
	
1	8	4 (50%)	45.9 (30.9-73.4)	27.8 (0.0,73.3)	
	
2	12	6 (50%)	61.3 (36.2)	59.5 (27.8,91.2)	

**Table 3:** Cost of Primary or Secondary PARPi Maintenance per Patient.

	BRCA Mutated Olpaparib	BRCA Mutated Niraparib	HRD^+^ Niraparib	HRD Niraparib
**Primary treatment OBS vs** (**TX**)	$1,380 ($512,307)	$1,090 ($254,150)	$820 ($225,400)	$540 ($93,150)
**Secondary treatment TX vs** (**OBS**)	$196,093 ($550)	$241,500 ($550)	$148,350 ($380)	$106,950 ($390)
**Total**	$197,473 ($512,857)	$242,590 ($254,700)	$149,170 ($225,780)	$107,490 ($93,540)

## Highlights:

- Primary and secondary maintenance PARPi therapy produced similar survivals.
- Twenty-nine percent of BRCA mutated patients with advanced-stage EOC did not recur within 3 years of follow-up.
- Delaying maintenance PARPi therapy to secondary instead of primary results in enormous cost savings.
